# Effect of Adjunctive Acupuncture on Pain Relief Among Emergency Department Patients With Acute Renal Colic Due to Urolithiasis: A Randomized Clinical Trial

**DOI:** 10.1001/jamanetworkopen.2022.25735

**Published:** 2022-08-09

**Authors:** Jian-Feng Tu, Ying Cao, Li-Qiong Wang, Guang-Xia Shi, Lian-Cheng Jia, Bao-Li Liu, Wei-Hai Yao, Xiao-Lu Pei, Yan Cao, He-Wen Li, Shi-Yan Yan, Jing-Wen Yang, Zhi-Cheng Qu, Cun-Zhi Liu

**Affiliations:** 1 International Acupuncture and Moxibustion Innovation Institute, Beijing University of Chinese Medicine, Beijing, China; 2 School of Acupuncture-Moxibustion and Tuina, Beijing University of Chinese Medicine, Beijing, China; 3 Emergency Department, Beijing Hospital of Traditional Chinese Medicine, Capital Medical University, Beijing, China; 4 Urinary Surgery, Beijing Hospital of Traditional Chinese Medicine, Capital Medical University, Beijing, China; 5 Office of Academic Research, Beijing Hospital of Traditional Chinese Medicine, Capital Medical University, Beijing, China

## Abstract

**Importance:**

Renal colic is described as one of the worst types of pain, and effective analgesia in the shortest possible time is of paramount importance.

**Objectives:**

To examine whether acupuncture, as an adjunctive therapy to analgesics, could accelerate pain relief in patients with acute renal colic.

**Design, Setting, and Participants:**

This single-center, sham-controlled, randomized clinical trial was conducted in an emergency department in China between March 2020 and September 2020. Participants with acute renal colic (visual analog scale [VAS] score ≥4) due to urolithiasis were recruited. Data were analyzed from October 2020 to January 2022.

**Interventions:**

After diagnosis and randomization, all patients received 50 mg/2 mL of diclofenac sodium intramuscular injection immediately followed by 30-minute acupuncture or sham acupuncture.

**Main Outcomes and Measures:**

The primary outcome was the response rate at 10 minutes after needle manipulation, which was defined as the proportion of participants whose VAS score decreased by at least 50% from baseline. Secondary outcomes included response rates at 0, 5, 15, 20, 30, 45, and 60 minutes, rescue analgesia, and adverse events.

**Results:**

A total of 115 participants were screened and 80 participants (66 men [82.5%]; mean [SD] age, 45.8 [13.8] years) were enrolled, consisting of 40 per group. The response rates at 10 minutes were 77.5% (31 of 40) and 10.0% (4 of 40) in the acupuncture and sham acupuncture groups, respectively. The between-group differences were 67.5% (95% CI, 51.5% to 83.4%; *P* < .001). The response rates of acupuncture were also significantly higher than sham acupuncture at 0, 5, 15, 20 and 30 minutes, whereas no significant difference was detected at 45 and 60 minutes. However, there was no difference between the 2 groups in rescue analgesia rate (difference 2.5%; 95% CI −8.8% to 13.2%; *P* > .99). No adverse events occurred during the trial.

**Conclusions and Relevance:**

These findings suggest that acupuncture plus intramuscular injection of diclofenac is safe and provides fast and substantial pain relief for patients with renal colic compared with sham acupuncture in the emergency setting. However, no difference in rescue analgesia was found, possibly because of the ceiling effect caused by subsequent but robust analgesia of diclofenac. Acupuncture can be considered an optional adjunctive therapy in relieving acute renal colic.

**Trial Registration:**

Chinese Clinical Trial Registry: ChiCTR1900025202

## Key Points

**Question:**

Can acupuncture, as an adjunctive treatment to analgesics, accelerate pain relief in patients with acute renal colic better than sham acupuncture?

**Findings:**

In this randomized clinical trial of 80 individuals, acupuncture plus intramuscular injection of diclofenac was safe and provided fast and substantial pain relief for patients with renal colic compared with sham acupuncture plus diclofenac in the emergency setting. However, no difference in rescue analgesia was found.

**Meaning:**

These findings suggest that acupuncture can be considered an optional adjunctive therapy in relieving acute renal colic.

## Teaser

This randomized clinical trial evaluates the effect of adjunctive acupuncture on pain relief among emergency department patients with acute renal colic due to urolithiasis.

## Introduction

Renal colic is a symptom complex, including excruciating flank or abdominal pain radiating to the groin, that has resulted in millions of emergency department visits worldwide.^1^ It is the most frequent symptom of the presence of a stone in the urinary system.^2^ Moreover, it is estimated that the prevalence of urinary calculi ranges from 1% to 20% worldwide because of geographical, climatic, ethnic, dietary, and genetic factors.^3,4^ It is described as one of the worst pains a patient can have. Therefore, effective analgesia in the shortest possible time is of paramount importance in the management of renal colic.^5,6^

According to the guidelines of the European Association of Urology,^4^ nonsteroidal anti-inflammatory drugs are the first choice to manage pain in patients with acute renal colic. Intramuscular injectable diclofenac sodium is commonly used in China because it is technically easy and fast to administer.^7,8^ In a previous study,^9^ Pain alleviation was observed with a mean (SD) of 18.64 (7.99) minutes after intramuscular diclofenac sodium injection. However, 37.5% of patients still experienced moderate or severe pain at 15 minutes.^10^ Therefore, an effective and available therapy is urgently needed to accelerate pain relief.

Acupuncture, as a complementary therapy from traditional Chinese medicine, is effective for the management of numerous types of pain conditions.^11,12^ Additionally, the white paper “Acupuncture’s Role in Solving the Opioid Epidemic”^13^ has recommended that acupuncture can safely, easily, and cost-effectively be incorporated into the emergency department to treat acute pain conditions. Moreover, several randomized clinical trials (RCTs)^14,15,16,17^ have shown that acupuncture results in a rapid pain score decrease within the first 10 minutes. However, it is not certain whether acupuncture, as an adjunctive treatment to analgesics, can accelerate pain relief in patients with acute renal colic. This trial aimed to investigate whether acupuncture, as adjunctive treatment to analgesics, can accelerate pain relief for patients with renal colic in the emergency department.

## Methods

### Study Design

This single-center, sham-controlled, RCT was performed at the emergency department of the Beijing Hospital of Traditional Chinese Medicine Affiliated with Capital Medical University. This trial was approved by the ethics committee of the Beijing Hospital of Traditional Chinese Medicine Affiliated with Capital Medical University and registered at Chinese Clinical Trial Registry. The protocol and statistical analysis plan have been previously published and are provided in Supplement 1.^18^ All eligible participants provided written informed consent before enrolling in the trial. This study followed the Consolidated Standards of Reporting Trials (CONSORT) reporting guideline.^19^

### Participants

The recruitment strategy primarily contained advertisements in outpatient clinics and the emergency department. Eligible participants were men or women aged 18 to 75 years who received a diagnosis of acute renal colic by radiography or ultrasonography examination (within the last 24 hours in the emergency department or other outpatient facility) according to the guidelines of the European Association of Urology^4^ and presented with moderate to severe renal colic on a visual analog (VAS) scale^20^ score of 4 or higher (range, 0-10, with higher scores indicating greater pain). The exclusion criteria were use of any analgesia in the last 6 hours; allergy to diclofenac sodium, morphine, or anisodamine; history of asthma, urticaria, or allergic rhinitis ascribed to acetylsalicylic acid or other drugs containing prostaglandin synthase inhibitors; congestive heart failure, acute ischemic heart disease, or peripheral vascular disease; acute cerebrovascular disease; increased intracranial pressure; kidney or liver failure; active digestive ulcer, pyloric obstruction, or intestinal obstruction; blood system diseases such as hemophilia or coagulation disorders; thrombocytopenia (<50×10^9^/L); use of anticoagulants; glaucoma, elevated intraocular pressure; serious adverse reactions to acupuncture; skin infection at acupuncture site; history of mental illness or substance abuse, or previous diagnosis of severe cognitive impairment (dementia); or pregnant or lactating.

### Randomization and Blinding

**Figure 1.**
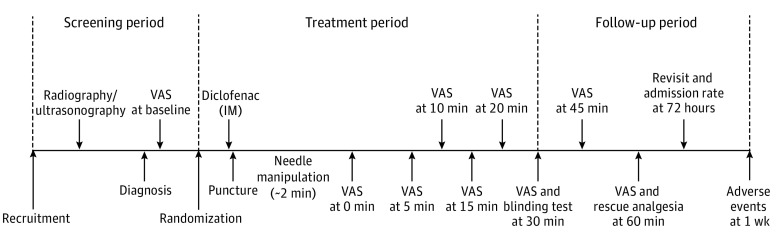
Study Procedures IM, intramuscular injection; VAS, visual analog scale.

**Figure 2.**
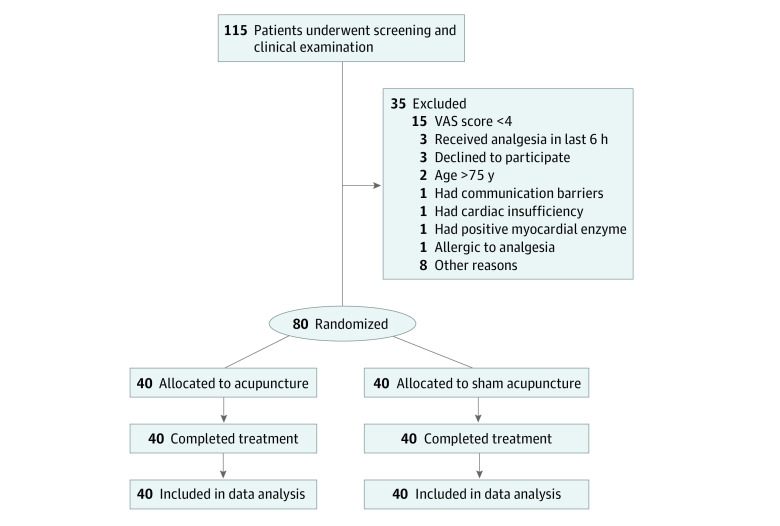
Patient Enrollment Flowchart VAS indicates visual analog scale.

Eligible patients were randomly assigned to the acupuncture group or sham acupuncture group in a 1:1 ratio. The blocked randomization sequence was generated by an independent statistician (not a coauthor of this article) using SAS statistical software version 9.4 (SAS Institute). Sealed envelopes were used to hide the group assignments, which were saved by a research assistant who did not take part in enrolling, treatment, or assessment. Envelopes were opened by acupuncturists when eligible patients were enrolled into the trial. Unmasked personnel included acupuncturists and research assistants responsible for the randomization module. All other study staff and researchers, including patients, outcome assessors, and the statistician, were masked to group. Patients were treated in a single treatment room. The randomization procedure is presented in Figure 1 and the patient enrollment flowchart is presented in Figure 2.

### Interventions

After the diagnosis of renal colic and randomization, patients received 50 mg/2 mL of diclofenac sodium (Guangdong Bangmin Pharmaceutical Co, Ltd) intramuscular injection according to the Chinese clinical pathway. Then 30-minute acupuncture or sham acupuncture was provided immediately. Both acupuncture and sham acupuncture were performed by licensed acupuncturists with at least 5 years of experience. All acupuncturists were trained on the location of acupoints and nonacupoints and the manipulation of needling before the trial began, and recorded the completion of the intervention. Sterile disposable acupuncture needles (length, 40 mm; diameter, 0.3 mm; Hwato) were used in both groups.

The bilateral *Yaotongdian* (EX-UE 7), 2 acupoints on each side of the hand, were used in the acupuncture group according to traditional Chinese medicine. These acupoints were developed from experience with clinical experts. Needles were inserted at an angle of 90° and a depth of 0.5 cun (about 8-10 mm). Twirling, lifting, and thrusting (needle manipulation) were performed for at least 30 seconds per acupoint to reach De qi sensation (soreness, numbness, distention, and heaviness). According to a search and analysis of traditional Chinese medicine reference books and modern acupuncture articles, acupoints with effects on alleviating acute renal colic were screened. After excluding these acupoints, nontreatment related acupoints on the forearm were extracted and locations 3 mm away from these nontreatment related acupoints were defined as nonacupoints that were used in the sham acupuncture group. To make the quantity of stimulus uniform between 2 groups, the same number of needles for sham acupuncture were used as in the acupuncture group. The 16 nonacupoints were randomly assigned to 8 subgroups by an independent statistician and were recorded in predetermined computer-randomized sealed envelopes. Each subgroup had 2 bilateral nonacupoints on the forearm. The patients in the sham acupuncture group were assigned into 1 of these 8 subgroups. Superficial skin penetration (1-4 mm in depth) at nonacupoints without De qi manipulations was performed in the sham acupuncture group. The location of acupoints and nonacupoints are shown in eTable 1 and the eFigure in Supplement 2. The similarities and differences between acupuncture and sham acupuncture groups are summarized in eTable 2 in Supplement 2.

If patients reported the severity of pain as higher than an 8 score on the VAS at 60 minutes after acupuncture treatment, rescue analgesia would be used. Rescue analgesia was 0.1 mg/kg intravenous morphine (Northeast Pharmaceutical Group Shenyang First Pharmaceutical Co, Ltd) and 10 mg intramuscular racanisodamine (TianJin KingYork Pharmaceutical Co, Ltd). Morphine is an opioid receptor agonist and is thought to have a strong analgesic effect. Racanisodamine is a type of muscarinic cholinergic antagonist and is recommended in Chinese guidelines^21^ to relieve smooth muscle spasms. Racanisodamine has been chosen as active control in several trials, and a response rate between 75.5% and 81.4% was found.^22,23^ No additional intravenous fluid was administered in the first 60 minutes after acupuncture treatment.

### Outcomes

The primary outcome was the response rate at 10 minutes after needle manipulation. The response rate was defined as the proportion of participants whose pain score on VAS reduced by at least 50% compared with baseline.^8,11^

Secondary outcomes included response rate at 0, 5, 15, 20, 30, 45, and 60 minutes after needle manipulation; the VAS at 0, 5, 10, 15, 20, 30, 45, and 60 minutes after needle manipulation; rescue analgesia at 60 minutes after needle manipulation; revisit and admission rate at 72 hours after needle manipulation; and adverse events at 1 week. Revisit and admission rate and adverse events were assessed by the trained researchers contacting the patients over the telephone. All other outcomes were evaluated face-to-face. Patients were shown a 10-cm line with the anchor “no pain” on one end and “most pain possible” on the other end. A chronograph was used to ensure accurate timing of the VAS measurements. Moreover, all patients were asked to guess whether they received acupuncture or sham acupuncture after acupuncture treatment for the blinding test.

### Statistical Analysis

Sample size calculation was determined by the previous literature^24^ and our clinical experience. The response rates in the acupuncture group and sham acupuncture group were expected to be 70% and 40%, respectively. The ratio between acupuncture group and sham acupuncture group was 1:1. A sample size of 80 patients (40 in each group) was estimated to have at least 80% power to detect difference of 30% between groups at a 2-sided significance level of 5% according to the formula. Because there was only 1 session of acupuncture treatment, we did not consider loss to follow-up.

For baseline characteristics, continuously distributed variables were described using mean (SD) or median (IQR); discrete variables were described by frequencies and percentages. The analysis was based on the intention-to-treat principle and included all randomized patients. Response rate, rescue analgesia, and revisit and admission rate were evaluated with the χ^2^ test or Fisher exact test. For VAS score, a comparison between groups was assessed by a mixed-effects model with repeated measurement analysis using corresponding scale scores at all time points as dependent variables, treatment as the main factor, treatment by time as interaction effect, the baseline value as a covariate, and a random intercept to model within-subject correlation. The difference of VAS at 10 minutes was estimated with analysis of covariance adjusting for baseline VAS as sensitivity analysis. Analyses were performed with SPSS statistical software version 21.0 (IBM) with 2-sided *P* < .05 considered significant. Data were analyzed from October 2020 to January 2022.

## Results

Between March 2020 and September 2020, a total of 115 participants were screened. Of them, 35 participants were excluded, and 80 (66 men [82.5%]; mean [SD] age, 45.8 [13.8] years) were randomized (recruitment rate of 69.6%). Forty participants were in the acupuncture group and 40 were in the sham acupuncture group. All patients completed the trial (Figure 2). The demographic characteristics at baseline are shown in Table 1.

**Table 1.** Participant Demographic and Baseline Characteristics

Variable	Participants, No. (%)	
	Acupuncture (n = 40)	Sham acupuncture (n = 40)
Age, mean (SD), y	46.7 (13.1)	44.8 (14.6)
Sex		
Female	7 (17.5)	7 (17.5)
Male	33 (82.5)	33 (82.5)
Weight, mean (SD), kg	74.8 (11.0)	72.1 (11.2)
Height, mean (SD), cm	170.3 (7.4)	171.1 (5.6)
History of previous urolithiasis	13 (32.5)	15 (37.5)
Chronic illness		
Hypertension	11 (27.5)	5 (12.5)
Type 2 diabetes	4 (10.0)	8 (20.0)
Hyperlipemia	6 (15.0)	4 (10.0)
Initial pain, mean (SD), visual analog scale score^a^	7.7 (1.6)	7.5 (1.5)
Time from attack to treatment, mean (SD), h	8.3 (11.8)	10.3 (14.5)
Heart rate, mean (SD), beats per min	80.8 (15.5)	80.0 (12.5)
Systolic blood pressure, mean (SD), mm Hg	138.1 (20.8)	137.4 (19.5)
Diastolic blood pressure, mean (SD), mm Hg	83.4 (13.4)	82.2 (14.0)
Temperature, mean (SD), °C	36.2 (0.5)	36.1 (0.5)
White blood cell count, mean (SD), cells/μL	9500 (2900)	10 100 (3600)
Creatinine, mean (SD), mg/dL	0.94 (0.17)	0.95 (.24)
Stone size, mm		
≤5	29 (72.5)	29 (72.5)
>5	11 (27.5)	11 (27.5)
Stone side		
Left	20 (50.0)	26 (65.0)
Right	20 (50.0)	14 (35.0)
Stone location, ureter		
Upper	8 (20.0)	9 (22.5)
Middle	6 (15.0)	8 (20.0)
Lower	26 (65.0)	23 (57.5)

SI conversion factors: To convert creatinine to micromoles per liter, multiply by 88.4; white blood cell count to cells ×10^9^/L, multiply by 0.001.

^a^ The visual analog scale has a range from 1 to 10, with higher scores indicating worse pain.

**Figure 3.**
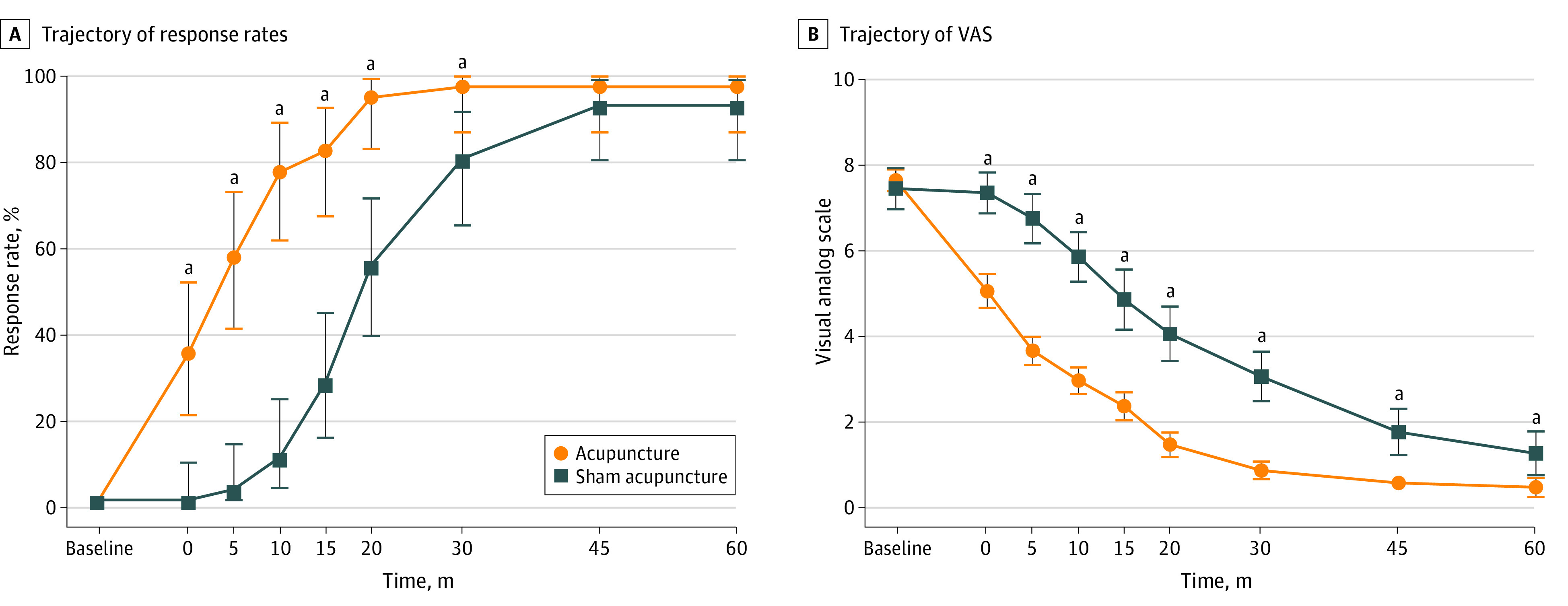
Trajectory of Response Rates and Visual Analog Scale (VAS) Scores Over Time in the Acupuncture and Sham Acupuncture Groups The time is calculated from the completion of needle manipulation. Bars show the 95% CI. The significant difference of response rate at 30 minute was calculated using Fisher exact test, whereas the 95% CI was calculated using Clopper and Pearson exact method. ^a^*P* < .05.

For the primary outcome, the acupuncture group benefited from higher response rates at 10 minutes than the sham acupuncture group (77.5% [31 of 40 patients] vs 10.0% [4 of 40 patients]; difference, 67.5%; 95% CI, 51.5%-83.4%; *P* < .001). The response rates of the acupuncture group were also significantly higher than the sham acupuncture group at 0, 5, 15, 20, and 30 minutes (97.5% [39 of 40 patients] vs 80.0% [32 of 40 patients]; difference, 17.5%; 95% CI, 4.2%-30.8%; P = .03), whereas no difference was detected at 45 and 60 minutes (97.5% [39 of 40 patients] vs 92.5% [37 of 40 patients]; difference, 5.0%; 95% CI, -4.5%-14.5%; P = .62). The changes in response rates at all assessment visits are presented in Table 2 and Figure 3A.

**Table 2.** Primary and Secondary Outcomes During the Study

Outcome and time	Acupuncture (n = 40)	Sham acupuncture (n = 40)	Difference (95% CI)	*P* value
Response rates, No. (%)^a^				
0 min^b^	14 (35.0)	0	35.0 (20.2 to 49.8)	<.001
5 min	23 (57.5)	1 (2.5)	55.0 (38.9 to 71.1)	<.001
10 min^c^	31 (77.5)	4 (10.0)	67.5 (51.5 to 83.4)	<.001
15 min	33 (82.5)	11 (27.5)	55.0 (36.8 to 73.2)	<.001
20 min	38 (95.0)	22 (55.0)	40.0 (23.2 to 56.8)	<.001
30 min	39 (97.5)	32 (80.0)	17.5 (4.2 to 30.8)	.03
45 min	39 (97.5)	37 (92.5)	5.0 (−4.5 to 14.5)	.62
60 min	39 (97.5)	37 (92.5)	5.0 (−4.5 to 14.5)	.62
VAS score, mean (SD)^d^				
0 min^b^	5.1 (2.5)	7.4 (1.5)	−2.3 (−3.2 to −1.4)	<.001
5 min	3.7 (2.1)	6.8 (1.8)	−3.1 (−4.0 to −2.3)	<.001
10 min	3.0 (2.0)	5.9 (1.8)	−2.9 (−3.8 to −2.1)	<.001
15 min	2.4 (2.1)	4.9 (2.2)	−2.5 (−3.4 to −1.6)	<.001
20 min	1.5 (1.8)	4.1 (2.0)	−2.6 (−3.4 to −1.7)	<.001
30 min	0.9 (1.3)	3.1 (1.8)	−2.2 (−2.9 to −1.5)	<.001
45 min	0.6 (1.2)	1.8 (1.7)	−1.2 (−1.9 to −0.6)	<.001
60 min	0.5 (1.4)	1.3 (1.6)	−0.8 (−1.4 to −0.2)	.02
Rescue analgesia rate at 60 min, No. (%)	1 (2.5)	0	2.5 (−8.8 to 13.2)	>.99
Revisit and admission rate, No. (%)	2 (5.0)	4 (10.0)	−5.0 − 23.1 to 14.1)	.68

Abbreviation: VAS, visual analog scale.

^a^ The response rate is defined as the proportion of participants whose VAS score decreased by at least 50% from baseline.

^b^ The time is calculated from the completion of needle manipulation.

^c^ Response rate at 10 minutes was the primary outcome; response rates at other time points, VAS, rescue analgesia rate, and revisit and admission rate were secondary outcomes.

^d^ The VAS is on a scale of 1 to 10, with higher scores indicating greater pain. The VAS was analyzed by a mixed effects model with repeated measurement using corresponding scale scores at all time points as dependent variable, treatment as the main factor, treatment by time as interaction effect, the baseline value as a covariate, and a random intercept to model within-subject correlation. VAS was significantly associated with the interaction of treatment and time, so *P* values at each time point are presented instead of the overall *P* value.

Table 2 presents VAS score over time from the mixed-effects model. VAS was significantly influenced by the interaction of treatment and time, so *P* values at each time point are presented. There was a significant reduction of VAS score in the acupuncture group compared with the sham acupuncture group at 0, 5, 15, 20, 30, 45, and 60 minutes (Figure 3B). In the sensitivity analysis, the between-group difference of VAS at 10 minutes was similar (difference, −3.2; 95% CI, −3.7 to −2.6; *P* < .001) (eTable 3 in Supplement 2). However, there was no statistically significant difference between the 2 groups on rescue analgesia rate (difference, 2.5%; 95% CI, −8.8% to 13.2%; *P* > .99) or revisit and admission rate (difference, −5.0%; 95% CI −23.1% to 14.1%; *P* = .68). No adverse reactions were found in the acupuncture and sham acupuncture groups. For the success of blinding (eTable 4 in Supplement 2), no difference was found between groups in the proportion of patients who guessed correctly what kind of acupuncture they had received (difference, 7.5%; 95% CI, −14.7% to 28.3%; *P* = .48).

## Discussion

In this RCT, we found that acupuncture as adjunctive treatment to analgesics provides faster and more substantial pain relief than sham acupuncture for patients with renal colic. There was a statistically significant difference between the 2 groups for the response rates of relieving pain at 10 minutes. Meanwhile, the benefit of acupuncture on relieving pain began when the needle manipulation completed and lasted until 45 minutes. However, no difference in rescue analgesia was found.

The results may have implications for the initial management of renal colic in emergency departments. Adjunctive acupuncture could offer fast and substantial relief from renal colic presentations in the emergency setting. Moreover, acupuncture of EX-UE 7 is therapy on a single acupoint, which does not need syndrome differentiation and is easily performed under emergency conditions. Thus, acupuncture might be considered an option for adjunctive treatment in relieving acute renal colic.

To our knowledge, no similar RCT has been found to evaluate acupuncture as an adjuvant treatment to analgesics for renal colic. However, we found 3 RCTs^14,16,17^ comparing acupuncture with analgesics in the treatment of renal colic. An RCT^17^ in China found that acupuncture had a more rapid analgesic onset compared with Avafortan (Asta-Werke Degussa Pharma Gruppe) (mean [SD] 3.14 [2.88] minutes vs 15.44 [7.55] minutes). We found a significant improvement in response rate and VAS score in the acupuncture group compared with the sham acupuncture group after needle manipulation (approximately 2 minutes), and the difference between groups reached its maximum at 5 minutes. This finding suggests that acupuncture combined with analgesics bring a faster onset time than analgesics alone.

A Tunisian trial^16^ showed a similar response rate at 60 minutes between the acupuncture (87%) and morphine (83%) groups. Our trial found the response rates at 30, 45, and 60 minutes were 97.5% in the acupuncture plus intramuscular diclofenac group, which was higher than acupuncture alone in the Tunisian trial. This suggests that acupuncture combined with diclofenac sodium may have a certain synergistic effect. A Qatari trial^8^ reported the response rate at 30 minutes was 68% in the diclofenac sodium group, which was lower than sham acupuncture plus diclofenac sodium (80%) in our trial. This may be related to the placebo effect of sham acupuncture.

For renal colic, previous studies^14^ have found that acupuncture was associated with rapid pain decrease, and the analgesic effect of diclofenac continued for a full 120 minutes after intake. Hence, our trial combined acupuncture with analgesic to explore a comprehensive therapy with fast and substantial effects for patients with renal colic. In addition, our trial selected shallow needling at 2 of 16 nonacupoints randomly as the sham control, which could reduce the impact of the psychological or physiological reactions of the patient and make the evaluation results more objective. Considering that the proportion of participants achieving a significant reduction in pain score is more patient-centric and more clinically relevant, our trial chose the response rate (decrease of at least 50% of VAS from baseline) as the primary outcome.

### Limitations

Our trial has several limitations. First, this is a single-center trial, which might introduce potential biases influencing the generalizability. However, compared with multicenter trials, the quality is more easily controlled and internal authenticity is better guaranteed in single-center trials. Second, both diclofenac and acupuncture were provided after diagnosis in this trial, but pain relief and other emergency measures should be offered before imaging to patients with severe pain.^4^ In the future, we will study whether it is appropriate to give patients acupuncture before imaging examination. Third, given the limited sample size, subgroup analysis was not performed by the kidney stone size and location. Fourth, acupuncturists could not be blinded and acupuncture was provided by experienced acupuncturists. It is unknown whether nonacupuncturist practices would produce the same results. Fifth, the data of pain relief beyond 60 minutes after the adjunctive acupuncture were not evaluated.

## Conclusions

The findings of this RCT suggest that acupuncture combined with intramuscular injection of diclofenac is safe and provides fast and substantial pain relief for patients with renal colic compared with sham acupuncture in the emergency setting. However, no difference in rescue analgesia was found, possibly because of the ceiling effect caused by subsequent but robust analgesia of diclofenac. Acupuncture can be considered an optional adjunctive therapy in relieving acute renal colic.

## Notes

Supplement 1.
Trial Protocol and Statistical Analysis Plan

Supplement 2. **eFigure.** Locations of Acupoints and Non-Acupoints **eTable 1.** Locations of Non-Acupoints in the Sham Acupuncture Group **eTable 2.** Similarities and Differences Between Acupuncture and Sham Acupuncture Groups **eTable 3.** Sensitivity Analysis of VAS with ANCOVA Adjusting for Baseline **eTable 4.** Evaluation of Blinding Test

Supplement 3.
Data Sharing Statement
