# Supplementary material for: Effect of Adjunctive Acupuncture on Pain Relief Among Emergency Department Patients With Acute Renal Colic Due to Urolithiasis: A Randomized Clinical Trial
Source: JAMA Netw Open. 2022 Aug 9;5(8):e2225735. doi: 10.1001/jamanetworkopen.2022.25735 (PMC9364130; doi:10.1001/jamanetworkopen.2022.25735)
Supplement: Supplement 1. — Trial Protocol and Statistical Analysis Plan [file jamanetwopen-e2225735-s001.pdf]

Acupuncture as adjunctive therapy for acute renal colic due to urolithiasis: a randomized controlled trial

Study Protocol
Final Version

Clinical site:

Beijing Hospital of Traditional Chinese Medicine, Capital Medical University, Dongcheng District, Beijing, China

Data management and statistical site:

International Acupuncture and Moxibustion Innovation Institute, School of Acupuncture-Moxibustion and Tuina, Beijing University of Chinese Medicine, Chaoyang District, Beijing, China

Data:

Original protocol date: April 21, 2019
Final protocol date: October 27, 2021

Contents

1 Committee composition 1
  1.1 Protocol committee 1
  1.2 Steering committee 1
  1.3 Data coordination committee 1
  1.4 Publication committee 1
2 Study contacts and organizations 1
  2.1 Study contacts 1
  2.2 Recruiting site 2
3 Background 2
4 Study hypotheses 3
5 Methodology 3
  5.1 Study design 3
  5.2 Randomization and blinding 3
  5.3 Sample size 3
  5.4 Participant recruitment, screening and group assignment 4
  5.5 Inclusion criteria 4
  5.8 Exclusion criteria 5
6 Outcome measurement 5
  6.1 Primary outcome 5
  6.2 Secondary outcomes 5
7 Interventions 6
  7.1 Acupuncture 6
  7.2 Sham acupuncture 6
8 Quality control 7
9 Statistical analysis 8
9 Protocol revision history 8
References 8

1 Committee composition

1.1 Protocol committee

Table 1 Protocol committee

Name	Affiliation	Email address
Cun-Zhi Liu	Beijing University of Chinese Medicine	lcz623780@126.com
Zhi-Cheng Qu	Beijing Hospital of Traditional Chinese Medicine	qzhch0824@163.com
Jian-Feng Tu	Beijing University of Chinese Medicine	tujianfeng1@126.com
Ying Cao	Beijing Hospital of Traditional Chinese Medicine	caoying7c@163.com
Lian-Cheng Jia	Beijing Hospital of Traditional Chinese Medicine	drjialiancheng@sina.com
Bao-Li Liu	Beijing Hospital of Traditional Chinese Medicine	liubaoli@bjzhongyi.com
Wei-Hai Yao	Beijing Hospital of Traditional Chinese Medicine	lyao@126.com
Guang-Xia Shi	Beijing University of Chinese Medicine	shiguangxia2008@126.com

1.2 Steering committee

Table 2 Steering committee

Name	Affiliation	Email address
Cun-Zhi Liu	Beijing University of Chinese Medicine	lcz623780@126.com
Zhi-Cheng Qu	Beijing Hospital of Traditional Chinese Medicine	qzhch0824@163.com
Jian-Feng Tu	Beijing University of Chinese Medicine	tujianfeng1@126.com
Ying Cao	Beijing Hospital of Traditional Chinese Medicine	caoying7c@163.com
Lian-Cheng Jia	Beijing Hospital of Traditional Chinese Medicine	drjialiancheng@sina.com
Jing-Wen Yang	Beijing University of Chinese Medicine	yangjw0626@126.com
Shi-Yan Yan	Beijing University of Chinese Medicine	yanshiyan0927@sina.com
Xiao-Lu Pei	Beijing Hospital of Traditional Chinese Medicine	luisa2013@163.com

1.3 Data coordination committee

Table 3 Data coordination committee

Name	Affiliation	Email address
Li-Qiong Wang	Beijing University of Chinese Medicine	wangliqiongwork@163.com
Yan Cao	Beijing University of Chinese Medicine	caoyan92cy@163.com
He-Wen Li	Beijing University of Chinese Medicine	1352686576@qq.com

1.4 Publication committee

Members of the publication committee include Cun-Zhi Liu, Zhi-Cheng Qu, Jian-Feng Tu and Ying Cao.

2 Study contacts and organizations

2.1 Study contacts

Principal investigator for grant

Cun-Zhi Liu, MD PhD

International Acupuncture and Moxibustion Innovation Institute, School of Acupuncture-Moxibustion and Tuina, Beijing University of Chinese Medicine; No. 11, Bei San Huan Dong Lu, Chaoyang District, Beijing 100029, China

Phone: +86 1053912201

Email: lcz623780@126.com

Zhi-Cheng Qu, MD, PhD
Beijing Hospital of Traditional Chinese Medicine Affiliated to Capital Medical University, No. 23 Meishuguanhou Street, Dongcheng District, Beijing 100010, China
Phone: +86 10 52176575
E-mail: qzhch0824@163.com

Principal investigator for trial

Cun-Zhi Liu, MD PhD
International Acupuncture and Moxibustion Innovation Institute, School of Acupuncture-Moxibustion and Tuina, Beijing University of Chinese Medicine; No. 11, Bei San Huan Dong Lu, Chaoyang District, Beijing 100029, China
Phone: +86 1053912201
Email: lcz623780@126.com

Zhi-Cheng Qu, MD, PhD
Beijing Hospital of Traditional Chinese Medicine Affiliated to Capital Medical University, No. 23 Meishuguanhou Street, Dongcheng District, Beijing 100010, China
Phone: +86 10 52176575
E-mail: qzhch0824@163.com

2.2 Recruiting site

Beijing Hospital of Traditional Chinese Medicine Affiliated to Capital Medical University

Zhi-Cheng Qu, MD, PhD
Beijing Hospital of Traditional Chinese Medicine Affiliated to Capital Medical University, No. 23 Meishuguanhou Street, Dongcheng District, Beijing 100010, China
Phone: +86 10 52176575
E-mail: qzhch0824@163.com

Ying Cao, MSc
Beijing Hospital of Traditional Chinese Medicine Affiliated to Capital Medical University, No. 23 Meishuguanhou Street, Dongcheng District, Beijing 100010, China
Phone: +86 10 52176575
E-mail: caoying7c@163.com

3 Background

Acute renal colic caused by urinary calculi (ARCUC) is described as acute unbearable paroxysmal pain in the lower back or upper abdomen, with or without hematuria, nausea, and vomiting.[1] Urinary stone disease was increasingly prevalent, with a lifetime risk of about 12% in men and 6% in women.[2] The prevalence of kidney stones in China was 6.4% (6.5% in men and 5.1% in women).[3] In the USA, more than one million patients visit the emergency department for the ARCUC every year.[4] It is described as one of the worst pains a patient could have and has a considerable impact on quality of life.

Pain relief is the primary goal in the management of patients with ARCUC.[1] NSAIDs offer effective sustained analgesia for ARCUC in the emergency department[5] and result in a lower need for rescue analgesia.[6] The 2017 update of the European Association of Urology (EAU) guidelines recommends

NSAIDs as the first-line analgesic.[1] However, its clinical application is partly limited for the increased risk of major coronary events which increase with dose and duration.[7, 8] Furthermore, the onset time of NSAIDs is relatively slow, with the time to peak plasma concentration of 10-30 minutes after intramuscular injection[9]. More than 30% patients did not achieve satisfactory relief of pain after had NSAIDs[5]. Adjunctive therapy with quick analgesia effect and less adverse event is needed for patients with ARCUC.

Acupuncture is a complementary therapy from traditional Chinese medicine, which has the advantages of quick analgesia.[10-11] A meta-analysis suggested that acupuncture may be a potential therapy for ARCUC.[12] However, to our knowledge, there is no randomized controlled trial (RCT) to measure the efficacy of acupuncture as adjunctive treatment to NSAIDs for ARCUC. This randomized, participant-blind, sham-controlled trial is designed to evaluate the efficacy and safety of acupuncture as adjunctive treatment to diclofenac for ARCUC.

4 Study hypotheses

The following hypotheses will be tested:

H1: There is a significant difference in the participants' response rate at 10 minutes between the acupuncture group and the sham acupuncture group

H0: There is no difference in the participants' response rate at 10 minutes between the acupuncture group and the sham acupuncture group

5 Methodology

5.1 Study design

This is a single-center, participant-blinded, parallel-group, randomized controlled study at Emergency Department in China. Participants with ARCUC will be randomized into acupuncture group or sham acupuncture group in a 1:1 ratio.

5.2 Randomization and blinding

The blocked randomization sequence will be computer-generated with the SAS 9.4 software by an independent professional statistician (Jing Hu, Beijing Hospital of Traditional Chinese Medicine, Capital Medical University), who is not involved in the implementation and statistical analysis of the trial. The sealed envelopes will be numbered in sequential order from 1 to 80 to hide the group assignments and be saved by a research assistant who does not take part in enrolling patients. When eligible patients are enrolled into the trial, envelopes will be successively opened by the clinical research coordinators who are responsible for enrolling the patients. Due to the responsibility of providing acupuncture and sham acupuncture, the acupuncturists will not be masked. Patients in the two acupuncture groups will be treated in a single treatment room and be blinded to which acupuncture method they would receive. In addition, outcome assessors, and statisticians who perform the statistical analyses will be blinded. The group assignments will be revealed after the statistical analysis is completed.

5.3 Sample size

In this study, the sample size was priori calculated. Based on the previous literature[13] and our clinical experience, the response rates in the acupuncture group and sham acupuncture group are expected to be 70% and 40%, respectively. The ratio between acupuncture group and sham acupuncture group was 1:1. A sample size of 80 patients (40 in each group) is estimated to have at least 80% power to detect difference between groups at a 2-sided significance level of 5% according to the formula: $n = \left[\frac{z_\alpha \sqrt{4\pi_c(1-\pi_c)} + z_\beta \sqrt{2\pi_1(1-\pi_1) + \pi_2(1-\pi_2)}}{\pi_1 - \pi_2} \right]^2$. Because there is only one session of acupuncture

treatment and almost no shedding, no loss to follow-up is considered. Thus, the recruitment goal was set at 80 patients.

5.4 Participant recruitment, screening and group assignment

Participants who are diagnosed as ARCUC according to the guideline of European Association of Urology will be recruited at Beijing Hospital of Traditional Chinese Medicine Affiliated to Capital Medical University.[1] The recruitment strategy will primarily contain advertisements on outpatient clinics, the emergency room and hospital social Internet media (WeChat). Written informed consent will be provided by each patient through research assistant before randomization. The evaluators will record the data on the electronic case report form (CRF) through the whole trial period. The participant flow was shown in Figure 1 and Figure 2.

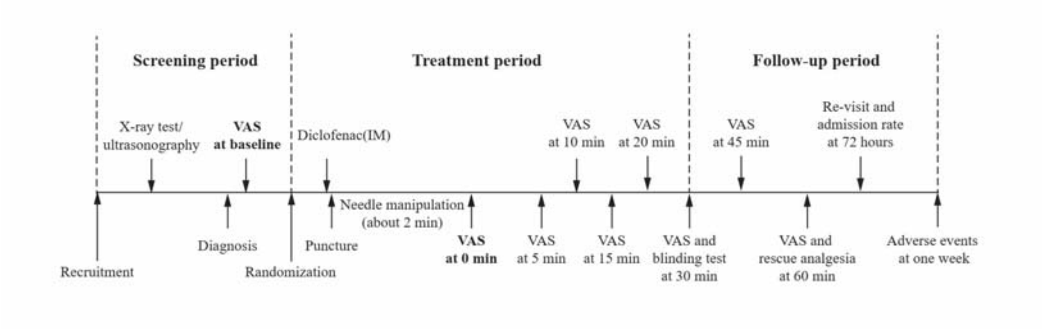

Figure 1 Procedure of treatment and visit

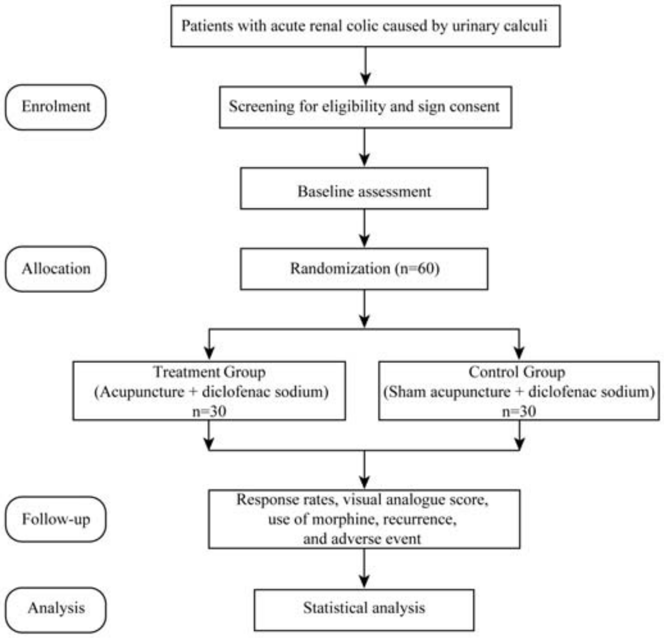

Figure 2 Flow diagram

5.5 Inclusion criteria

Participants will be included in the study if they meet the following criteria.

1. Diagnosed as ARCUC according to the guideline of European Association of Urology (2017)[1]
2. Aged 18-75 years (either sex)

3. Pain intensity of 4 or more out of 10 on a visual analogue scale (VAS)[14]

4. Written informed consent

5.8 Exclusion criteria

Participants will be excluded from the study if they met the following criteria.

1. Use of any analgesia in the last 6 hours

2. Allergic to diclofenac sodium, morphine, or anisodamine; history of asthma, urticaria or allergic rhinitis ascribed to acetylsalicylic acid or other drugs containing prostaglandin synthase inhibitors

3. Congestive heart failure, acute ischemic heart disease, or peripheral vascular disease; acute cerebrovascular disease, increased intracranial pressure; renal or liver failure

4. Active digestive ulcer, pyloric obstruction, or intestinal obstruction

5. Blood system diseases: such as hemophilia, coagulation disorders in patients; Thrombocytopenia (< $50*10^9$/L); using anticoagulants

6. Glaucoma, elevated intraocular pressure

7. Serious adverse reactions to acupuncture; skin infection at acupuncture site

8. History of mental illness or substance abuse, or have severe cognitive impairment

9. Pregnant or lactating.

6 Outcome measurement

For participants with unilateral osteoarthritis, the knee affected will be assessed throughout the entire study. For bilateral osteoarthritis, the most painful knee at baseline will be the one assessed throughout the entire study[15].

6.1 Primary outcome

The primary outcome was the response rate at 10 minutes after needle manipulation. The response rate was defined as the proportion of participants whose pain score on VAS reduces at least 50% compared with baseline.[5]

6.2 Secondary outcomes

1. Total pain

~~The response rate will also be measured at weeks 4, 16, and 26 after randomization.~~

The total pain will be defined by the area under the curve during the 60 minutes.[15] The pain will be assessed using a VAS[14] with scores ranging from 0 to 10 at baseline and after 0, 5, 10, 15, 20, 30, 45, and 60 minutes. The bigger of the area under the curve indicates worse pain.

2. Response rate at other times

The proportion of participants achieving significant pain reduction will also be measured after 0, 5, 15, 20, 30, 45, and 60 minutes.

3. Remedial analgesia

The number of patients who receive intravenous morphine and intramuscular racanisodamine will be recorded after 60 minutes.

4. Re-visit and admission rate

The numbers of patients who re-visit the emergency department or are hospitalized will be evaluated during 72 hours.

5. Blinding assessment

All patients will be asked to guess whether they receive acupuncture or sham acupuncture after acupuncture treatment to measure the patient-blinding effects.

6. Adverse events

All adverse events will be recorded by outcome assessors during 7 days after treatment. Based on

the potential relationship between needling and adverse events, adverse events will be categorized as treatment-related or not.

7 Interventions

Patients in both acupuncture group and sham acupuncture group will receive 50 mg/2 mL diclofenac sodium intramuscular injection after randomization (Guangdong Bangmin Pharmaceutical Co, LTD). Both acupuncture and sham acupuncture will be performed by the licensed doctors of traditional Chinese medicine with at least 5 years of experience. Meanwhile, acupuncture will be performed by the licensed doctors of traditional Chinese medicine who have been trained how to locate acupoints and non-acupoints, puncture, and manipulate needles before the trial. Sterile disposable stainless steel acupuncture needles (length: 40 mm, diameter: 0.3 mm; Hwato, Suzhou, China) will be used. Both acupuncture and sham acupuncture treatment will only consist of 1 session treatment with 30 minutes. Needles will be removed if the patients suffer from any adverse events (AEs). Patients will receive 0.1mg/kg intravenous morphine (Northeast Pharmaceutical Group Shenyang First Pharmaceutical Co, LTD) and 10mg intramuscular racanisodamine (Tianjin KingYork Pharmaceutical Co, LTD) if they report the severity of pain more than 8 points on the VAS. No additional intravenous fluid will be administered in the first 60 minutes after administration of the diclofenac sodium.

7.1 Acupuncture

Patients allocated to the acupuncture group will be punctured at the pre-specified acupoints. According to the theory of traditional Chinese medicine and clinical experience, bilateral Yaotongdian (EX-UE 7) will be used. According to the National standard of the People's Republic of China, EX-UE 7 will contain two points on the dorsum of the hand. The one is between the second and the third metacarpal bones, and the other is between the fourth and the fifth metacarpal bones. These two points are of the same distance to the metacarpophalangeal joints and the transverse crease of the wrist. The localization of EX-UE 7 is exhibited in Figure 3. Four needles will be used for per patient, and the depth of needle insertion will be 8-10 mm. Manipulations of twirling, lifting, and thrusting will be performed on all needles for at least 30s to reach De qi (a compositional sensation including soreness, numbness, distention, and heaviness), which is believed to be an essential component for acupuncture efficacy.

7.2 Sham acupuncture

A superficial skin penetration (1–4 mm in depth) at non-acupoints will be performed in the sham acupuncture group, without needle manipulation for De qi. Based on the search and analyses of traditional Chinese medicine reference books and acupuncture modern articles, the acupoints with effects on alleviating ARCUC or pain have been screened. After excluding these acupoints, 16 points without effects on ARCUC and pain are extracted and the locations 3 mm apart from these 16 acupoints are defined as non-acupoints, which are used in the sham acupuncture group. The locations of these non-acupoints are shown in Table 4 and Figure 3. To make the quantity of stimulus uniform between two groups, the same number of needles for sham acupuncture will be same to those in verum acupuncture. The 16 non-acupoints will be randomly assigned to 8 subgroups and will be recorded in predetermined computer-made randomization sealed envelopes. Each subgroup has bilateral 2 non-acupoints on the arms. The patients in the sham acupuncture group will be assigned into 1 of these 8 subgroups. This method to define non-acupoints in sham acupuncture has been used in previous acupuncture trials[16].

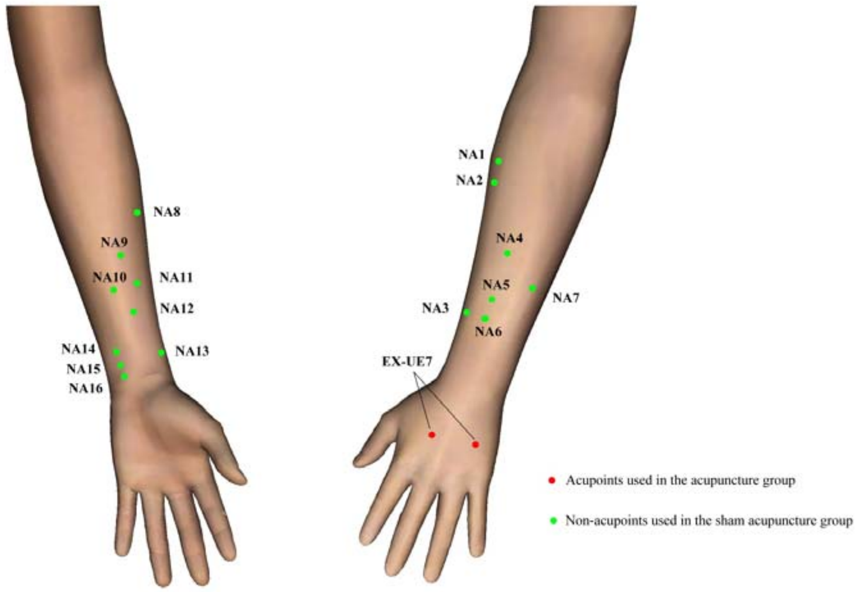

Figure 3 Locations of acupoints and non-acupoints

Table 4 Locations of non-acupoints in SA group

Subgroup	Non-Acupoints	Locations
1	NA 1	3 mm lateral to the Shanglian (LI9) horizontally
	NA 3	3 mm lateral to the Pianli (LI6) horizontally
2	NA 4	3 mm lateral to the Sidu (TE9) horizontally
	NA 16	3 mm lateral to the Yinxi (HT6) horizontally
3	NA 6	3 mm lateral to the Zhigou (TE6) horizontally
	NA 8	3 mm lateral to the Kongzui (LU6) horizontally
4	NA 12	3 mm lateral to the Jianshi (PC5) horizontally
	NA 2	3 mm lateral to the Xialian (LI8) horizontally
5	NA 7	3 mm lateral to the Zhizheng (SI7) horizontally
	NA 11	3 mm lateral to the Erbai (EX-UE2) horizontally
6	NA 5	3 mm lateral to the Sanyangluo (TE8) horizontally
	NA 13	3 mm lateral to the Jingqu (LU8) horizontally
7	NA 9	3 mm lateral to the Ximen (PC4) horizontally
	NA 15	3 mm lateral to the Tongli (HT5) horizontally
8	NA 14	3 mm lateral to the Lingdao (HT4) horizontally
	NA 10	3 mm internal to the Erbai (EX-UE2) horizontally

8 Quality control

Both paper files and electronic documents will be preserved for at least 5 years after publication. If readers have any questions, they can contact the corresponding author for access to the original data. Patient information will remain anonymous, including name, ID number and telephone number. The protocol will be reviewed and revised by experts in acupuncture, emergency, urinary surgery, methodology and statistics. We will perform a pre-specified standard operating procedure, which

includes screening patients, improve relevant inspection, intramuscular injection of diclofenac, acupuncture, filling out the CRF, assessing outcomes and data management. On-site monitoring will be adopted in this trial per three months. The ethics committee of Beijing Hospital of Traditional Chinese Medicine Affiliated to Capital Medical University will audit trial conduct per 12 months.

9 Statistical analysis

Patients' baseline characteristics will be summarized based on groups. Continuous variables will be described using the mean (standard deviation), or the median (interquartile range) if the normality assumption is violated. Student's t text or Wilcoxon rank sum test (if normality is violated) will be used for comparison of continuous variables among the two groups. Categorical variables will be described using the frequency (percentage) and compared using the chi-squared test.

For the primary comparison, the chi-squared test will be used for the response rate (the proportion of participants whose pain reduced ≥ 50% compared with baseline). For the secondary outcomes, Student's t test, chi-squared test, Fisher's exact test or the Wilcoxon rank sum test will be used to test the difference of the outcomes including the total pain, remedial analgesia, re-visit and admission rate, blinding assessment, and adverse events, between groups according to the distribution of variables. There is no interim analysis or additional analysis in this trial.

All efficacy analyses will be performed using the intention-to-treat set, which includes all randomized patients. Missing data will be dealt with the last observation carried forward (LOCF). All analyses will be performed using SPSS version 23.0 (IBM SPSS Statistics, New York, USA). The level of significance will be established at $\alpha < 0.05$ with a two-sided test.

9 Protocol revision history

1. The time points of 0 minute and 1 minute were changed as baseline and 0 minute, respectively.
2. The mixed effects model was used to evaluate the between-group differences of the total pain during the trial instead of Student's t test, because it is more conservative.
3. The difference of VAS at 10 minutes was evaluated with analysis of covariance (ANCOVA) adjusting for baseline VAS as sensitivity analysis according to the advice of reviewers.
4. The depth of puncture in acupuncture was 8-10 mm in this trial. The original depth of 10-15mm was a calculation error.

References

1. Türk C, Petřík A, Sarica K, Seitz C, Skolarikos A, Straub M, et al. EAU Guidelines on Diagnosis and Conservative Management of Urolithiasis. Eur Urol. 2016;69:468-74.
2. Curhan GC. Epidemiology of stone disease. Urol Clin North Am. 2007;34:287-93.
3. Zeng G, Mai Z, Xia S, Wang Z, Zhang K, Wang L, et al. Prevalence of kidney stones in China: an ultrasonography based cross-sectional study. BJU Int. 2017;120:109-16.
4. Ghani KR, Roghmann F, Sammon JD, Trudeau V, Sukumar S, Rahbar H, et al. Emergency department visits in the United States for upper urinary tract stones: trends in hospitalization and charges. J Urol. 2014;191:90-6.
5. Pathan SA, Mitra B, Straney LD, Afzal MS, Anjum S, Shukla D, et al. Delivering safe and effective analgesia for management of renal colic in the emergency department: a double-blind, multigroup, randomised controlled trial. Lancet. 2016;387:1999-2007.
6. Pathan SA, Mitra B, Cameron PA. A Systematic Review and Meta-analysis Comparing the Efficacy of Nonsteroidal Anti-inflammatory Drugs, Opioids, and Paracetamol in the Treatment of Acute Renal Colic. Eur Urol. 2018;73:583-95.

7. Krum H, Swergold G, Gammaitoni A, Peloso PM, Smugar SS, Curtis SP, et al. Blood pressure and cardiovascular outcomes in patients taking nonsteroidal antiinflammatory drugs. Cardiovasc Ther. 2012;30:342-50.
8. Coxib and traditional NSAID Trialists' (CNT) Collaboration, Bhala N, Emberson J, Merhi A, Abramson S, Arber N, et al. Vascular and upper gastrointestinal effects of non-steroidal anti-inflammatory drugs: meta-analyses of individual participant data from randomised trials. Lancet. 2013;382:769-79.
9. Dyloject (diclofenac sodium) Injection. https://www.accessdata.fda.gov/drugsatfda_docs/nda/2014/022396Orig1s000TOC.cfm. Accessed 5 August 2020.
10. Lin LL, Wang LQ, Yang JW, Tu JF, Wang TQ, Zou X, et al. Researches status on time-effect of acupuncture. Zhongguo Zhen Jiu. 2019;39:565-70.
11. Lee YH, Lee WC, Chen MT, Huang JK, Chung C, Chang LS. Acupuncture in the treatment of renal colic. J Urol. 1992;147:16-8.
12. Hong JH, Huang JL, Lu ZK. Acupuncture therapy for calculous renal colic: a meta-analysis. Asia-Pacific Trad Med. 2017;13:59-62.
13. Ju BJ, Niu LL. Analysis of therapeutic effect of acupuncture at Neiguan (PC 6) and Zusanli (ST 36) on acute renal colic. Zhongguo Zhen Jiu. 2012;32:975-8.
14. Hjermstad MJ, Fayers PM, Haugen DF, Caraceni A, Hanks GW, Loge JH, et al. Studies comparing Numerical Rating Scales, Verbal Rating Scales, and Visual Analogue Scales for assessment of pain intensity in adults: a systematic literature review. J Pain Symptom Manage. 2011;41:1073-93.
15. Wang LP, Zhang XZ, Guo J, Liu HL, Zhang Y, Liu CZ, et al. Efficacy of acupuncture for acute migraine attack: a multicenter single blinded, randomized controlled trial. Pain Med. 2012;13:623-30.17. Sio TT, Le-Rademacher JG, Leenstra JL, Loprinzi CL, Rine G, Curtis A, et al. Effect of Doxepin Mouthwash or Diphenhydramine-Lidocaine-Antacid Mouthwash vs Placebo on Radiotherapy-Related Oral Mucositis Pain: The Alliance A221304 Randomized Clinical Trial. JAMA. 2019;321:1481-90.
16. Wang LP, Zhang XZ, Guo J, Liu HL, Zhang Y, Liu CZ, et al. Efficacy of acupuncture for migraine prophylaxis: a single-blinded, double-dummy, randomized controlled trial. Pain. 2011;152:1864-71.
