# Supplementary material for: Effect of Adjunctive Acupuncture on Pain Relief Among Emergency Department Patients With Acute Renal Colic Due to Urolithiasis: A Randomized Clinical Trial
Source: JAMA Netw Open. 2022 Aug 9;5(8):e2225735. doi: 10.1001/jamanetworkopen.2022.25735 (PMC9364130; doi:10.1001/jamanetworkopen.2022.25735)
Supplement: Supplement 2. — eFigure. Locations of Acupoints and Non-Acupoints eTable 1. Locations of Non-Acupoints in the Sham Acupuncture Group eTable 2. Similarities and Differences Between Acupuncture and Sham Acupuncture Groups eTable 3. Sensitivity Analysis of VAS with ANCOVA Adjusting for Baseline eTable 4. Evaluation of Blinding Test [file jamanetwopen-e2225735-s002.pdf]

Supplemental Online Content

Tu JF, Cao Y, Wang LQ, et al. Effect of adjunctive acupuncture on pain relief among emergency department patients with acute renal colic due to urolithiasis: a randomized clinical trial. *JAMA Netw Open*. 2022;5(8):e2225735.
doi:10.1001/jamanetworkopen.2022.25735

eFigure. Locations of Acupoints and Non-Acupoints
eTable 1. Locations of Non-Acupoints in the Sham Acupuncture Group
eTable 2. Similarities and Differences Between Acupuncture and Sham Acupuncture Groups
eTable 3. Sensitivity Analysis of VAS with ANCOVA Adjusting for Baseline
eTable 4. Evaluation of Blinding Test

This supplemental material has been provided by the authors to give readers additional information about their work.

© 2022 Tu JF et al. *JAMA Network Open.*

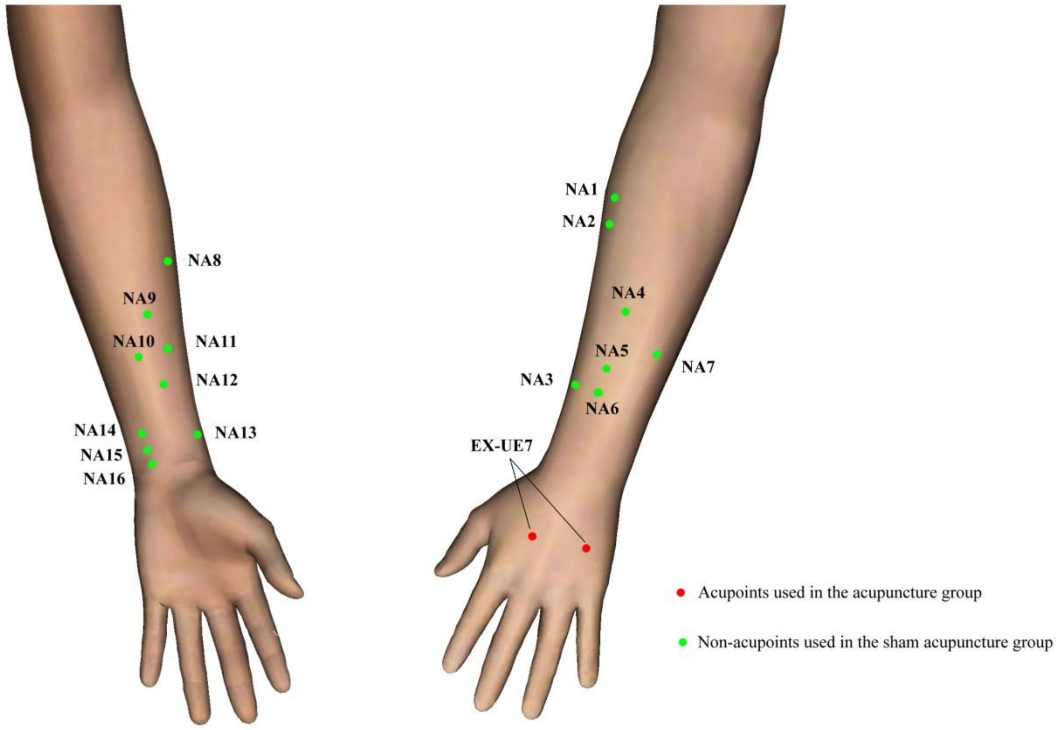

eFigure 1. Locations of acupoints and non-acupoints

© 2022 Tu JF et al. *JAMA Network Open.*

eTable 1. Locations of non-acupoints in the sham acupuncture group

Subgroup	Non-Acupoints	Locations
1	NA 1	3 mm lateral to the Shanglian (LI9) horizontally
	NA 3	3 mm lateral to the Pianli (LI6) horizontally
2	NA 4	3 mm lateral to the Sidu (TE9) horizontally
	NA 16	3 mm lateral to the Yinxi (HT6) horizontally
3	NA 6	3 mm lateral to the Zhigou (TE6) horizontally
	NA 8	3 mm lateral to the Kongzui (LU6) horizontally
4	NA 12	3 mm lateral to the Jianshi (PC5) horizontally
	NA 2	3 mm lateral to the Xialian (LI8) horizontally
5	NA 7	3 mm lateral to the Zhizheng (SI7) horizontally
	NA 11	3 mm lateral to the Erbai (EX-UE2) horizontally
6	NA 5	3 mm lateral to the Sanyangluo (TE8) horizontally
	NA 13	3 mm lateral to the Jingqu (LU8) horizontally
7	NA 9	3 mm lateral to the Ximen (PC4) horizontally
	NA 15	3 mm lateral to the Tongli (HT5) horizontally
8	NA 14	3 mm lateral to the Lingdao (HT4) horizontally
	NA 10	3 mm internal to the Erbai (EX-UE2) horizontally

NA, non-Acupoint.

© 2022 Tu JF et al. *JAMA Network Open.*

eTable 2. Similarities and differences between acupuncture and sham acupuncture groups

Items	Acupuncture group	Sham acupuncture group
Similarities		
Area of points stimulated	Upper limb	
Number of session	1	
Number of acupoints/non-acupoints	2	
Number of needles	4	
Needle retention, min	30	
Specification of needles	Length: 40 mm; diameter: 0.3 mm	
Differences		
Kinds of points stimulated	Acupoints	Non-acupoints
Region of points stimulated	Forearm	Dorsum of the hand
Location of points stimulated	EX-UE 7	3 mm away from non-treatment related acupoints (two of LI9, LI6, TE9, HT6, TE6, LU6, PC5, LI8, SI7, EX-UE2, TE8, LU8, PC4, HT5, and HT4)
Depth of penetration, mm	about 10	1-4
De qi	Yes	No

© 2022 Tu JF et al. *JAMA Network Open.*

eTable 3. Sensitivity analysis of VAS with ANCOVA adjusting for baseline

Time	Acupuncture (n=40)	Sham acupuncture (n=40)	Difference (95% CI)	*P*
0 min	5.1 (2.5)	7.4 (1.5)	-2.5 [-3.0, -2.0]	<0.001
5 min	3.7 (2.1)	6.8 (1.8)	-3.3 [-3.9, -2.8]	<0.001
10 min	3.0 (2.0)	5.9 (1.8)	-3.2 [-3.7, -2.6]	<0.001
15 min	2.4 (2.1)	4.9 (2.2)	-2.7 [-3.4, -2.0]	<0.001
20 min	1.5 (1.8)	4.1 (2.0)	-2.7 [-3.4, -2.0]	<0.001
30 min	0.9 (1.3)	3.1 (1.8)	-2.3 [-2.9, -1.7]	<0.001
45 min	0.6 (1.2)	1.8 (1.7)	-1.3 [-1.9, -0.7]	<0.001
60 min	0.5 (1.4)	1.3 (1.5)	-0.9 [-1.5, -0.2]	0.007

Abbreviations: VAS: visual analogue scale; ANCOVA, analysis of covariance.
Data were presented as mean (SD).

© 2022 Tu JF et al. *JAMA Network Open.*

eTable 4. Evaluation of blinding test

Guess	Acupuncture (n=40)	Sham acupuncture (n=40)	*P*
Correct	6 (15.0)	3 (7.5)	0.48
Unknown	34 (85.0)	37 (92.5)	
Incorrect	0	0	

© 2022 Tu JF et al. *JAMA Network Open.*
