# Supplementary material for: Effect of Adjunctive Acupuncture on Pain Relief Among Emergency Department Patients With Acute Renal Colic Due to Urolithiasis: A Randomized Clinical Trial
Source: JAMA Netw Open. 2022 Aug 9;5(8):e2225735. doi: 10.1001/jamanetworkopen.2022.25735 (PMC9364130; doi:10.1001/jamanetworkopen.2022.25735)
Supplement: Supplement 3. — Data Sharing Statement [file jamanetwopen-e2225735-s003.pdf]

# Data Sharing Statement

Tu. Effect of Adjunctive Acupuncture on Pain Relief Among Emergency Department Patients With Acute Renal Colic Due to Urolithiasis. *JAMA Netw Open*. Published August 09, 2022. doi:10.1001/jamanetworkopen.2022.25735

**Data**
**Data available:** Yes
**Data types:** Deidentified participant data
**How to access data:** The deidentified participant data are available upon reasonable request until 2 years after publication, via the corresponding author [e-mail, lcz623780@126.com]. The data can be made available to researchers who provide a methodologically sound proposal in their request.
**When available:** With publication

**Supporting Documents**
**Document types:** None

**Additional Information**
**Who can access the data:** Researchers whose proposed use of the data has been approved.
**Types of analyses:** For any purpose or for a specified purpose
**Mechanisms of data availability:** after approval of a proposal
